# Psychosocial Impact of Infertility Diagnosis and Conformity to Gender Norms on the Quality of Life of Infertile Spanish Couples

**DOI:** 10.3390/ijerph21020158

**Published:** 2024-01-30

**Authors:** Lidia Bueno-Sánchez, Tamara Alhambra-Borrás, Alfonso Gallego-Valadés, Jorge Garcés-Ferrer

**Affiliations:** Polibienestar Research Institute, Universitat de València, 46022 València, Spain; tamara.alhambra@uv.es (T.A.-B.); alfonso.gallego@uv.es (A.G.-V.); jordi.garces@uv.es (J.G.-F.)

**Keywords:** infertility, psychosocial well-being, quality of life, gender norms

## Abstract

Epidemiological data show that human reproductive disorders are a common problem worldwide, affecting almost one in six people of reproductive age. As a result, infertility has been identified by the World Health Organization as a public health disease. Reproductive problems can take a heavy toll on the psychosocial well-being of couples suffering from infertility. This is especially true for women, who tend to be the ones who undergo the most treatment. The main objective of the present study is to find out whether a sex-based infertility diagnosis influences the quality of life of couples with infertility. Also, we aim to find out whether the degree of adherence to gender norms influences their quality of life. A cross-sectional study was conducted using the Fertility Quality of Life Questionnaire (FertiQoL) and the Conformity to Feminine and Masculine Norms Inventories in a sample of 219 infertile Spanish couples (438 participants). The results show that, in all cases, regardless of the degree of conformity to gender norms and whether the infertility diagnosis was of female or male origin, women have lower scores on the self-perceived quality of life. This suggests that being female is already a psychosocial risk factor when assessing the psychosocial consequences of infertility.

## 1. Introduction

Infertility affects 17.5% of the world’s adult population, approximately one in six people, according to a report by the World Health Organization [1]. This implies that the prevalence of infertility among couples of reproductive age ranges from 12.6% to 17.8% worldwide, with relatively higher prevalence rates in some regions, such as the Americas, the Western Pacific, Africa, and Europe [2].

Infertility is considered as a public health problem that affects the psychosocial level of those who suffer from it [3]. Assisted reproduction technique (ART) interventions are a handicap for the general health of those who undergo them [2]. In addition, it often entails a large financial cost for couples [4]. Despite this, ARTs have become the first intervention to solve conception problems. Therefore, being infertile and undergoing fertility treatments are conditions that affect different areas of the lives of infertile individuals and couples [5]. Reactions to infertility include shock, sadness, depression, anger, frustration, and loss of self-esteem; especially, women are affected, as they receive most of the treatments and interventions on their bodies [6,7,8]. For this reason, the quality of life (QoL) of both women and men can be seriously affected.

Infertility and its treatment are significantly related to aspects of relational life, psychological well-being, and psychological correlates, such as anxiety and depression [9,10,11]. Moreover, the scientific literature has shown that the psychological state of couples undergoing fertility treatments significantly influences the outcome of the treatment [12]. All this seems to show the imperative need to identify and understand the risk factors for adverse psychology in order to be able to provide person-centered care [12,13,14,15,16]. Consequently, assessing the components of infertile couples’ quality of life and identifying the factors that affect it are important—not only to improve their health and psychophysical balance, but also to design more favorable and efficient infertility treatment programs, as well as to improve adherence to them [17].

QoL is a concept that aims to understand the well-being, whether of a population or individual, regarding both positive and negative elements within the entirety of their existence at a specific point in time. For example, common facets of QoL include personal health (physical, mental, and spiritual), relationships, education status, work environment, social status, wealth, a sense of security and safety, freedom, autonomy in decision-making, social belonging, and their physical surroundings [18]. It is a very broad concept that is influenced in a complex way by the physical health of people’s psychological state, their level of independence and their social relationships, as well as their relationship with the essential elements of their environment [19]. According to the reviewed studies, the factors that determine the quality of life in infertile people are: sexuality, mental health, social relationships, age, the duration of infertility, communication, educational level, marital relationship, medical history, and economic considerations [8,17,20].

Historically, women’s gender identity has been constructed on the basis of motherhood and femininity. There is a strong association between productive capacity and female gender identity [21]. As a result of social and economic transformations and the increased presence of feminism, the roles associated with motherhood and fatherhood have been redefined during the 21th century [22]. However, there are still gaps in the widespread social conception of motherhood as one of women’s main aspirations [23]. As a consequence of the traditional association between women and reproduction, men have also been affected by the weight of gender in relation to infertility [24,25,26]. Stereotypical masculinity rejects vulnerability in men, promotes the appearance of toughness and emotional control, and minimizes the need for help from others; so, the diagnosis of infertility has profound implications for men’s identity/role as a part of a couple and has often been associated with impotence or lack of virility. Consequently, it is not surprising that infertility and its diagnosis can have a different impact on men and women. Sex and gender influence the diagnosis, course, treatment, and outcomes of many illnesses, as well as the access to and acceptability of psychosocial and health care [27,28]. To measure any of these aspects, both concepts—sex and gender—must be clearly defined.

Gender is a social construct that refers to roles, activities, and behaviors and encompasses a wide range of identities beyond male, female, and intersex [29]. It focuses on the implications of society’s psychosocial framework for society in relation to the norm of being a man or a woman [29]. The gendered approach to infertility allows visualizing the multiple ways in which gender constructions shape identity, socialization processes, and inequality, which affect the health and quality of life of infertile women and men.

Sex, on the other hand, refers to the biological and physiological characteristics that define infertile women and men, the physiological characteristics that define humans as male, female, or intersex humans. It is defined as the unique physical make-up of males and females due to chromosomal, reproductive, and hormonal differences [30].

Given the above, the present study aims to investigate the effect of sex-specific diagnosis (male versus female fertility factors) on the quality of life of heterosexual infertility couples.

In order to answer this objective, the present study looked for differences that could be distinguished between the QoL of men and women based on the assignment of a sex-specific diagnosis, i.e., does the QoL of men and women depend on whether infertility is attributed to a male or female factor?

To this end, differences in QoL between the subgroups of the study sample (male, female; fertile and infertile) were studied. Finally, the relationship between quality of life and conformity to gender norms was explored. This provided further insights into the risk factors affecting the psychological well-being and quality of life of infertile couples.

## 2. Materials and Methods

### 2.1. Study Design and Participants

The present study conducted a cross-sectional comparative research using a study sample of heterosexual couples attending the Assisted Reproduction Unit to determine whether differences could be distinguished between male and female QOL based on the assignment of a sex-specific diagnosis (male vs. female infertility factors).

The sample was selected by convenience strictly to exclude other intervening variables (single-parent families, same-sex couples, etc.). The sample consisted of a total of 219 heterosexual couples (219 men and 219 women).

The inclusion criteria for the research project were: having difficulty to conceive during at least twelve months, being over 18 years of age, being in a heterosexual relationship, and having enough knowledge of the Spanish language to be able to sign the informed consent.

Data collection was conducted at the Assisted Reproduction Unit of the Hospital Universitario y Politécnico (HUP) La Fe in Valencia (Spain) between January 2018 and August 2020. All participants in this study provided a written informed consent. The design and administration of the informed consent was approved by the ethics committee of the HUP La Fe (reference: 2018/0383). The participants included in the study completed the self-administered questionnaires in a physical space set up in the Assisted Reproduction Unit, administered by a member of the research team and delivered on the same day. No incentives were offered to participate in the study.

### 2.2. Study Variables and Instruments

On the one hand, to study QoL, the Spanish version of the Fertility Quality of Life Questionnaire (FertiQoL) provided by its authors (http://sites.cardiff.ac.uk/fertiqol/files/2015/02/fertiqol-Spanish.pdf, accessed on 27 October 2023) [31,32] was used in the study. The FertiQoL questionnaire was developed to assesses the impact of infertility problems in several areas: personal quality of life, interpersonal quality of life, treatment-related quality of life, and overall satisfaction with physical health and quality of life. The instrument consists of 24 items, divided into two modules: a general module and treatment module. The general module assesses quality of life in 4 subscales: emotional (feelings and individual experiences associated with fertility problems, such as depression or envy); mind–body (physical and cognitive symptoms, such as a lack of concentration or tiredness); relational (aspects related to the relationship with a partner); and social (measures the impact on social interactions, support, etc.). The treatment module assesses the perception of treatment in two subscales: treatment environment and treatment tolerability. Each item is assessed on a five-point Likert-type scale ranging from 0 to 4, with a total score ranging from 0 to 100. Higher scores indicated a better fertility-specific quality of life [31].

To investigate gender norm conformity, the participants were administered the adapted Spanish version of the Conformity to Feminine and Masculine Norms Inventories (Conformity to Feminine Norms Inventory (CFNI-23) and the Conformity to Masculine Norms Inventory (CMNI-23)) [33,34,35]. These inventories are two instruments that allow measuring different aspects of femininity and masculinity, respectively. They integrate a response method, based on behaviors, affects, and cognitions, that allows conceiving, from a broad perspective, the ways in which women and men adhere to a norm [33,34,35]; in particular, the CFNI-23 contains 23 items that assess conformity with seven female gender norms: invest in appearance, care for children, domestic, modesty, nice in relationships, romantic relationship, sexual fidelity, and thinness. The CMNI-23 also contains 23 items that deal with the following male gender norms: winning, emotional control, risk-taking, violence, power over women, dominance, playboy, self-reliance, primacy of work, disdain for homosexuals, and pursuit of status. In both CFNI-23 and CMNI-23, answers are provided on a 4-point Likert-type scale ranging from 0 (strongly disagree) to 3 (strongly agree). The total score of the questionnaire can range from 0 to 69. The higher the score, the higher the agreement [36].

The sociodemographic and clinical characteristics of the participants were also collected in order to obtain a complete profile of the participants as well as obtaining relevant data for our research, such as the type of diagnosis based on sex.

### 2.3. Data Analysis

First, descriptive analyses were carried out to explore the data, in particular, to present the characteristics of the study participants by means of frequency, percentages, means, and standard deviation.

The statistical procedures included: (1) a *t*-test for continuous data to compare the differences between the study sample subgroups (male, female, fertile, and infertile) and a paired *t*-test to explore the intra-couple’s differences (differences between the male and female of the same couple); and (2) a correlation analysis to explore the relation among QoL and gender norm conformity.

All statistical analyses were performed using R (version 4.0.2, R Foundation for Statistical Computing, Vienna, Austria). A *p*-value < 0.05 was considered statistically significant [37].

## 3. Results

The study sample consisted of a total of 438 participants undergoing fertility treatments: 219 heterosexual couples (219 men and 219 women). The average age of the total sample was 36.2 years, most participants were married (78.3%), and the mean of the relationship duration was 10.7 years. Regarding educational attainment, most of the sample completed at least secondary education (43.4% attained a secondary education and 36.1% had a university degree). The analysis of the employment situation showed that most participants (84.5%) were employed.

Regarding the clinical variables, fertility issues were due to a male factor in 49.3% of the cases and in 50.7% of the cases due to female factor. The couples presented a mean duration of difficulty to conceive of 4.5 years (ranging from 1 to 15 years). About the treatment, 8.7% were undergoing artificial insemination, ARTs in 85.2% of the cases (including in vitro fertilization (IVF), intracytoplasmic sperm injection (ICSI), and embryo transfer), and ICSI with preimplantation genetic testing in 5.3% of participants. Data on the type of treatment were missing for 0.9% of the sample. On average, the duration of the treatment was 1.9 years (ranging from 1 to 11 years) and the mean number of cycles was 2.8 (ranging from 1 to 13). Finally, most participants reported not having a previous pregnancy (74.2%), not having given birth (89.5%), and not having suffered an abortion (80.1%). All these sociodemographic and clinical data are explained in detail in Table 1.

Regarding the first specific objective, from the 219 men composing the total male sample, 107 (48.9%) had a male factor infertility diagnosis and 112 (51.1%) had a female factor diagnosis. Of the 219 women, 110 (50.2%) were diagnosed with fertility issues due to a female factor and 109 (49.8%) due to a male factor.

To look for differences that could be distinguished between men’s and women’s QoL based on the assignment of a gender-specific diagnosis, a *t*-test was conducted between the men and women who were infertile (the type of diagnosis coincides with sex, i.e., males with male factor infertility diagnosis) and fertile (diagnosis does not coincide with sex). Table 2 and Table 3 present the *t*-test results.

Table 2 shows the differences between men and women according to their fertility status (fertile or infertile). Statistically significant intergroup differences were observed (*p* < 0.01) between both men and women who were fertile and men and women who were infertile. No statistically significant difference was found between men (fertile vs. infertile) and between the women’s sample (fertile vs. infertile).

In Table 3, the differences between men and women based on the assignment of a sex-specific diagnosis (male vs. female factor infertility diagnosis) are presented. The results of the *t*-test between these groups showed, again, differences between men and women (*p* < 0.01). On the one hand, statistically significant intergroup differences were found for the sample with male factor infertility diagnosis. On the other hand, significant differences were encountered between men and women with female factor infertility diagnosis.

Based on these results, it can be concluded that differences between men and women (intergroup differences) were in all cases relevant and statistically significant. Women had a worse QoL compared to men, independently of the diagnosis, and even when women were fertile, they had lower scores in the FertiQoL than men. Intragroup differences were found to be not statistically significant; however, men with a male factor infertility diagnosis presented a worse QoL than the men who were fertile (FertiQoL means of 74.69 vs. 76.3, respectively), and the same happened with women. The women with a female factor infertility diagnosis showed a poorer QoL in comparison to the women who were fertile (FertiQoL means of 68.17 vs. 68.82, respectively).

Therefore, the fertility status (fertile vs. infertile) and the sex-specific diagnosis (male vs. female) does not show significant differences in the perception of QoL in men or women. However, it does when these comparisons are established between men and women, showing that the variable gender may be a relevant factor influencing the perception of QoL in men and women with fertility issues.

To explore the differences in QoL between the male and female of the same couple (intra-couple’s differences), an intraclass correlation coefficient (ICC) analysis and a paired *t*-test were conducted. These tests were carried out with 205 of the couples who had no missing data (note that ICC analysis requires a balanced design and, thus, it deletes cases/observations with missing values). The QoL’s ICC between the members of the same couple showed a low degree of convergence with the FertiQoL score (r = 0.536 t = 9.06, df = 203 (*p* < 0.01); ICC = 0.458 F(204, 31.8) = 3.22, *p* = 0.000); however, the scores were moderately proportional between the two members of the couple (the higher the score of one member, the higher the score of the other).

The results of the intra-couple’s differences indicate statistically significant differences between the men and women of the same couple. In couples in which the man was fertile and the woman was infertile, the differences were (d = 7.605 (CI 95%: 5.34, 9.87), t = 6.669, df = 101, *p* = 0.000), and in couples in which the man was infertile and the woman fertile, (d = 6.396 (CI 95%: 3.81, 8.98), t = 4.908, df = 102, *p* = 0.000).

Regarding the second specific objective, Pearson’s correlation analyses were conducted to explore the relation between QoL (FertiQoL) and gender norm conformity (CMNI and CFNI). The results of these analyses show that a negative correlation between QoL and gender norm conformity was present in all the study groups. In particular, for men with an infertility diagnosis, a negative correlation between FertiQoL and CFNI was found (r(103)= −0.07 (CI 95% = −0.26, 0.12), t = −0.750, *p* = 0.454), as well as for fertile men, (r(104) = −0.11 (CI 95% = −0.3, 0.08), t = −1.172, *p* = 0.243). Among the women, a negative correlation was found for women with an infertility diagnosis (r(107)= −0.02 (CI 95% = −0.21, 0.17), t = −0.237, *p* = 0.812) and for fertile women (r(107) = 0.00 (CI 95% = −0.19, 0.19), t = −0.031, *p* = 0.974).

According to these results, the QoL (FertiQoL scores) is higher among men compared to women, showing again that gender may play a relevant role in QoL perception. However, there is no evidence that conformity with gender norms plays a relevant role in the perception of QoL, even though it can be observed that a higher conformity with the dominant gender norms (feminine norms if female and masculine norms if male) indicates lower scores in QoL.

## 4. Discussion

In light of these results, it can be concluded that women undergoing fertility treatments have a worse QOL compared to men, regardless of the diagnosis and gender conformity. Although the results are in line with those obtained by other authors [38,39], this conclusion should be carefully considered, as further research involving a larger and more heterogeneous sample is needed. In addition, differences according to the sex of diagnosis (male or female infertility factor) were analyzed in this sample, since the object of study was the effect of diagnosis according to sex and conformity to gender norms on the quality of life of infertile heterosexual couples. This research has some limitations: (1) we did not analyze differences according to the etiology of infertility, the severity of infertility, or the effects of PCOS-type syndrome, which could interfere with the quality of life scores; (2) as we did not cross-check the results with sociodemographic variables, we do not know whether these variables interfere with quality of life [39,40].

In line with the results obtained by other authors, our results argue that there is a clear sex- and gender-dependent difference in the self-perceived quality of life [6,7,8,9,10,11]. Women reported the lowest quality of life in all cases. Moreover, the study went a step further by providing evidence that women had a poorer quality of life than men even when they were not the infertile partner. This indicates that being a woman is already a psychosocial factor to be taken into account when analyzing the quality of life of people with infertility problems.

Although the degree of conformity to gender norms does not seem to influence the perceived quality of life of infertile couples, in all cases, women have a worse quality of life scores. That is, women tend to have a lower self-perceived quality of life, regardless of their conformity to gender norms. It is also worth noting the limitations of the questionnaire in terms of nonconformity with gender norms. The instrument in its Spanish version shows an acceptable consistency; so, the results obtained could vary if another instrument or research method, such as in-depth interviews, were used. Nevertheless, it is the most commonly used instrument for this type of research [40]. This study demonstrates that the sex and gender perspective should be present and guide psychosocial and health studies as differentiated measurement indicators. Consequently, sex/gender variables treated independently in research on the impact of infertility on people’s quality of life are important in studies and programs based on comprehensive person-centered care [12,13,14,15,16].

For future research, a comparative analysis of the impact of infertility in same-sex couples is recommended. Also, comparative studies should be conducted between heterosexual couples who receive psychological support during infertility treatment and couples who undergo treatment without psychological care. Special attention should be paid to the results obtained in relation to women’s quality of life. Longitudinal studies that take into account these variables and others, such as cultural environment or religious background, are also recommended. Likewise, the design of studies with mixed methodologies that incorporate in-depth interviews with couples undergoing assisted reproduction techniques is strongly recommended.

Thus, the present study poses an important question for future research: what is the underlying reason for women reporting a worse quality of life than men in all cases when it comes to infertility? Perhaps studies of a psychological and social nature could shed light on this.

## 5. Conclusions

Based on the premise that women report a lower quality of life than men, mental or social health professionals may seek to design programs that target women in the pre-treatment phases of infertility by designing programs that adapt to their psychosocial needs during the stages and cycles of treatment. More knowledge is needed on the differences in quality of life and conformity to intragroup gender norms in couples with infertility in the form of studies analyzing the self-perceived quality of life of pregnant mothers or single-parent families with fertility problems. The aim is to improve the quality and impact of psychosocial interventions. This would improve the quality of the intervention and may even improve intervention outcomes.

Knowledge of the self-perception of the quality of life of people with infertility, as well as the analysis of gender in the couple, can help the health or social professional to design more efficient support programs that improve the well-being of people with non-elective infertility through integrated person-based care.

## Figures and Tables

**Table 1:** Sociodemographic and clinical characteristics of the study participants (*n* = 438).

Characteristics	Study Participants (*n* = 500)
Age, mean (SD)	36.2 (4.5)
Sex, *n* (%)	
Female	219 (50.0%)
Male	219 (50.0%)
Marital status, *n* (%)	
Married	343 (78.3%)
Not married	95 (21.7%)
Relationship duration (years), mean (SD)	10.7 (5.0)
Educational attainment, N (%)	
No primary education	12 (2.7%
Primary education	78 (17.8%)
Secondary education	190 (43.4%)
University	158 (36.1%)
Employment situation, N (%)	
Employed	370 (84.5%)
Not in the labor force	68 (15.5%)
Fertility issues’ duration (years), mean (SD)	4.5 (2.2)
Fertility issues’ cause, N (%)	
Male factor	216 (49.3%)
Female factor	222 (50.7%)
Type of treatment, N (%)	
Artificial insemination	38 (8.7%)
ART (IVF, ICSI, and embryo transfer)	373 (85.2%)
ICSI with preimplantation genetic testing	23 (5.3%)
Missing information	4 (0.9%)
Treatment duration (years), mean (SD)	1.9 (1.3)
Number of cycles, mean (SD)	2.8 (2.2)
Previous pregnancy, N (%)	
Yes	97 (22.1%)
No	325 (74.2%)
Previous births, N (%)	
Yes	20 (4.5%)
No	392 (89.5%)
Previous abortions, N (%)	
Yes	71 (16.2%)
No	351 (80.1%)

Note. ART = Assisted reproductive treatment; IVF = In vitro fertilization; ICSI = Intracytoplasmic sperm injection.

**Table 2:** Differences between men’s and women’s QoL based on the fertility status.

		Fertile	Infertile
**Men**	N	112	107
	FertiQoL mean (SD)	76.3 (10.61)	74.69 (11.81)
	Intragroup differences (men)	t = 1.0616 (df = 217)	
**Women**	N	109	110
	FertiQoL mean (SD)	68.82 (14.72)	68.82 (14.72)
	Intragroup differences (women)	t = 0.35212 (df = 217)	
**Intergroup differences**		t = 4.3421 ** (df = 219)	t = 4.3421 ** (df = 219)

** *p*-value < 0.01.

**Table 3:** Differences between men’s and women’s QoL based on the assignment of a sex-specific diagnosis.

		Male Factor Infertility Diagnosis	Female Factor Infertility Diagnosis
**Men**	N	107	112
	FertiQoL mean (SD)	74.69 (11.81)	76.3 (10.61)
**Women**	N	109	110
	FertiQoL mean (SD)	68.82 (14.72)	68.82 (14.72)
**Intergroup differences**		t = 3.2284 ** (df = 214)	t = 5.1992 ** (df = 220)

** *p*-value < 0.01.

## Data Availability

Bueno-Sánchez, Lidia (2024), “Dataset_FertiQol_Gender_Couples”, Mendeley Data, V1, https://doi.org/10.17632/tk9b9xccvm.1.
